# Exploring Experiences and Designing Guidance for Involving and Engaging Children and Young People in James Lind Alliance Priority Setting Partnerships

**DOI:** 10.1111/hex.70195

**Published:** 2025-03-02

**Authors:** Laura Postma, Faith Gibson, Jasmijn Z. Jagt, Karijn Aussems, Helen Barrett, Malou Luchtenberg, Casper G. Schoemaker, Rachel K. Temple, Susie Aldiss

**Affiliations:** ^1^ University of Groningen, Beatrix Children's Hospital University Medical Center Groningen Groningen the Netherlands; ^2^ School of Health Sciences University of Surrey Guildford UK; ^3^ Centre for Outcomes and Experience Research in Children's Health Illness and Disability (ORCHID), Great Ormond Street Hospital for Children NHS Foundation Trust London UK; ^4^ Department of Paediatric Gastroenterology, Emma Children's Hospital Amsterdam University Medical Centre Amsterdam the Netherlands; ^5^ Department of Ethics, Law and Humanities Amsterdam UMC (Location VUmc) Amsterdam The Netherlands; ^6^ The James Lind Alliance, National Institute for Health and Care Research, School of Healthcare Enterprise and Innovation University of Southampton Southampton UK; ^7^ Department of Pediatric Rheumatology Wilhelmina Children's Hospital University Medical Centre Utrecht Utrecht The Netherlands; ^8^ The McPin Foundation London UK

**Keywords:** children and young people, guideline, James Lind Alliance, patient and public involvement and engagement, priority setting partnerships, research agendas, tips

## Abstract

**Introduction:**

Setting research priorities together with children and young people (CYP) in James Lind Alliance (JLA) Priority Setting Partnerships (PSPs) is an example of involving CYP from the beginning of the research process. In these PSPs, CYP can be involved in steering groups, surveys, focus groups and the final priority‐setting workshop. The success of JLA PSPs is evident, but it is important to note that specific guidance has not been available on how to involve CYP aged under 18 years. We aimed to collect experiences and tips from CYP who have taken part in a priority‐setting exercise and to work with them to develop guidance for the JLA on involving CYP in PSPs.

**Methods:**

This project was conducted in the United Kingdom and the Netherlands and was coordinated by the JLA. The project was overseen by a project group consisting of eight people, this group invited CYP who had participated in previous JLA PSPs. A workshop in the Netherlands and in the United Kingdom was organised. Throughout the workshops, the primary focus was exploring the first‐hand experiences of CYP. We developed ‘tips’ on how best to involve CYP. The project was conducted between May 2023 and February 2024.

**Results:**

Four CYP were involved in the UK workshop and three in the Dutch workshop. One young person had to cancel and was interviewed separately. Tips are divided into three categories, to reflect the different elements of the JLA method: steering group, surveys and the final priority‐setting workshop including preparations and evaluations. Tips centred on three themes: making participants feel comfortable, making the process accessible and making the involvement of CYP meaningful.

**Discussion:**

This project provides a list of 29 tips for involving CYP in JLA PSPs and guides future partnerships seeking to involve CYP. It guides teams during the planning, execution and completion of the project.

**Patient and Public Contribution:**

Children and young people with experience in JLA PSPs shared their tips with us for involving CYP in future PSPs. We examined their perceptions of what worked well and their constructive insights into areas for refinement in involving CYP in developing a research agenda.

## 1 Introduction

There has been an increasing effort in the involvement of children and young people (CYP) in health and care research [1, 2]. The involvement of CYP in research is more than just an effort to tick the box of inclusivity; it is a meaningful acknowledgment of their authority and a recognition of their inherent capacity to influence the research landscape [3]. Reasons to involve CYP in research are numerous and summarised here. First, the Convention on the Rights of the Child (UNCRC) states that CYP have the right to express their views on all matters affecting them [4]. Second, CYP have consistently expressed their desire to be involved in research that pertains to issues impacting upon them [5]. Third, the involvement of CYP impacts the research, it ensures that research is more relevant, accessible and meaningful [6]. Fourth, involvement contributes to young people's personal development [7] and strengthens them on their journey toward self‐actualization. It helps them gain insights into themselves, and better understand others, both within the context of research and in their daily lives [8, 9]. CYP acquire research skills and gain an understanding of the research process [10], gain confidence [6, 9, 10, 11, 12] and feel empowered [9, 13].

Children and young people can be involved in all research areas including prioritisation, design, data collection and analysis, dissemination and reporting of findings [14, 15]. Previous studies suggest that it is best to involve children in the research process as early as possible [16], starting with the identification of research questions or priorities. In 1995, Chalmers stated that increased involvement of patients and the public in shaping research agendas would likely result in a more open‐minded approach about which research questions are worth addressing [17]. Later he found a mismatch between the research questions rated important by patients and the public and those addressed in research, resulting in research waste [15]. This led to Chalmers and colleagues developing James Lind Alliance (JLA) Priority Setting Partnerships (PSPs) [18]. Following the JLA method people with lived experience, carers and health and care professionals collaboratively identify and prioritise unanswered questions that they agree are the most important [19]. The JLA method typically involves bringing together evidence uncertainties or unanswered questions via a survey from as many contributors as possible. Then the survey responses need to be reviewed, interpreted, sorted and turned into a list of indicative questions for research. These questions need to be checked and verified as true uncertainties. The interim prioritisation is the stage where the long list of questions for research is reduced to a shorter list by undertaking a second survey to prioritise the unanswered questions. The last stage is the final priority‐setting workshop to rank all the shortlisted research uncertainties to identify the Top 10 priorities in that topic area [20].

The JLA is a UK‐based nonprofit‐making initiative. The PSPs have the potential to impact the people who take part in them, the areas they set priorities in, and the research that is funded. The JLA has demonstrated its effectiveness in establishing over 150 PSPs, some of the recent Top 10 research priorities consider paediatric irritable bowel syndrome, first‐time soft tissue knee injuries, pemphigus and pemphigoid and concussion (https://www.jla.nihr.ac.uk/top-10-priorities/). Developing research agendas together with CYP is an example where they can be involved from the beginning of the research process. Recent studies have shown that more and more CYP are involved in PSPs [21, 22, 23]. To our knowledge, 30 research agendas have been developed together with CYP, of those, 19 were developed using the JLA method [7]. While the success of the JLA PSPs is evident, specific guidance on including those under 18 years old remains unavailable. Although previous guidance has addressed the involvement of CYP in research planning, design and execution [24], it has not specifically focused on their role in research priority setting. Meanwhile, an increasing number of PSPs are aiming to include CYP in all aspects of the JLA process and have turned to the JLA for advice on how to achieve this effectively.

## 2 Aim

To collect experiences and tips from young people who have taken part in a priority‐setting exercise and to work with them to develop guidance for the JLA on involving CYP in PSPs. We hope that this guidance will encourage teams who plan to do a PSP to involve CYP. This ensures that the resulting priorities reflect more widely the views of those with lived experience.

## 3 Methods

### 3.1 Project Group

The recent Children's Cancer PSP successfully involved CYP in identifying and prioritising questions [25, 26]. The JLA approached two researchers (S.A. and F.G.) from the coordinating team to write guidance to share with PSPs about involving CYP in a priority setting. Representatives from other PSPs who had successfully involved CYP [27, 28, 29] were invited to join a guidance development group to share their methods and experiences. Early on in our discussions, the group decided that it was important to hear from CYP about their experiences of taking part in a PSP and include their voices in the guidance about what works best for them, to ensure that the guidance would be a relevant and effective tool in developing research agendas together with CYP.

This project was conducted in the United Kingdom and the Netherlands and was coordinated by the JLA. The project was overseen by a group consisting of eight people including researchers with experience in coordinating a JLA PSP (F.G., S.A., C.S., J.J.), a youth involvement facilitator with experience in developing research agendas with CYP using the JLA method (R.T.), a researcher who evaluated developing research agendas with CYP (L.P.), a critical friend (K.A.) and a JLA representative (H.B.). The project group played a crucial role in shaping the overall plans. They collaborated in contacting CYP who had previously been involved in research priority‐setting exercises and determined the overarching topics explored during the workshops. The project group met six times online between May 2023 and February 2024.

### 3.2 Sampling and Inviting

Members of the project group invited CYP they had previously worked with in JLA PSPs. Inclusion criteria for CYP were: CYP with experience in developing a research agenda who were below the age of 18 years at the time of participating in the development of the research agenda and fluent in English or Dutch. We did not predetermine a sample size, as this was not a research study. We were aware that only a small number of CYP were involved in JLA PSPs. Nonetheless, we decided to form two separate small groups to ensure that all CYP participating in this project had the opportunity to share their perspectives. The CYP were contacted by email or WhatsApp depending on how the project group members usually communicated with them. The United Kingdom and the Netherlands were selected as study locations because these countries had completed the majority of JLA PSPs and had effectively adapted the JLA methodology to successfully involve CYP.

### 3.3 Design

The collaboration with CYP took place in two distinct groups. To ensure that the young people could speak freely in their native language, the first workshop was Dutch‐spoken and organised in the Netherlands and the second was English‐spoken and organised in the United Kingdom. Throughout the sessions, the primary focus was on exploring the first‐hand experiences of CYP. This exploration spanned a spectrum, encompassing their perceptions of what worked well and their constructive insights into areas for refinement in involving CYP in developing a research agenda, to develop ‘tips’ on how best to involve them. The CYP were thanked for their contribution with a voucher of €25/£20 and a sweet treat was sent to them after the workshop.

This article reports on developing guidance together with CYP. This was not a research project in which CYP were respondents, but they were involved as research partners, thus no approval from an ethics perspective was required. The CYP gave their consent to be mentioned by their own name as they wanted their contributions to be recognized. They are named as co‐authors on the JLA guidance document and are named in the acknowledgments section of this manuscript.

#### 3.3.1 The Dutch Workshop

The Dutch workshop was conducted in November 2023, led by L.P. and J.J. Four young people were invited. The workshop was conducted online and lasted 60 min. It started with a brief explanation of its purpose and the young people and researchers introduced themselves. Then the young people were asked to use Mentimeter to write down some words that were associated with the JLA according to them. These words were explored to start the discussion about the JLA method (e.g., what did you mean when you used the word, ‘interesting’, can you tell us more about that?). The young people were subsequently asked to share tips about the different aspects of the JLA method. This was an informal discussion, based on their experiences and how they felt CYP could best be involved in each aspect. Prompts for this discussion included what they found effective or less effective, and their recommendations for improving the less effective aspects. The session was audio‐recorded and transcribed by the researchers involved in the workshops.

#### 3.3.2 The UK Workshop

The UK workshop was conducted in February 2024 and led by F.G. and S.A. Six young people were invited to participate. Four young people participated in the workshop. The workshop was conducted online and lasted 90 min (including a short break). As in the Dutch workshop, it started with an explanation of the purpose of the meeting and introductions. Using Mentimeter, the young people were asked to write down three words to describe their experiences of the JLA workshop they had attended. These words were explored to start the discussion about their experiences. The young people were then asked to share tips for involving CYP in the different elements of JLA priority setting (steering group, survey and the final workshop). This was an informal discussion, similar to the Dutch workshop. The session was recorded and automatically transcribed through Microsoft Teams.

### 3.4 Data Analysis

One lead of each workshop reviewed the recording and transcripts, paying attention to insights shared by young people. Subsequently, these insights were documented and synthesized into a comprehensive list to encompass all their suggestions. While striving to maintain fidelity to the expressions of the CYP, their contributions were paraphrased rather than written verbatim. Our aim was to translate the input from the CYP into clear, actionable tips that were written concisely. Subsequently, the tips were returned to the CYP for validation, to ascertain whether the meaning had been retained. We sought to condense raw data into a summary format using a general inductive approach. The results of the two workshops were presented by the leads of the workshops to members of the project group. The next step in the process was for all the tips to be displayed according to the different JLA elements. Once this had been completed, overarching themes were identified. We used the GRIPP2 checklist to report PPI in our research [30].

## 4 Results

### 4.1 Young People

Table 1 provides details of the young people involved in the guidance development. Three young people, aged between 13 and 19, took part in the Dutch workshop. They were involved in the final priority‐setting workshop of the PSP about paediatric inflammatory bowel disease (IBD) in the Netherlands. Unfortunately, one of the young people was unable to attend the workshop due to illness and had to cancel. We called her a week later to seek her views. She was aged 21 and had been involved in the final workshop of the PSP about Glycogen‐Storage Disease in the Netherlands. Four young people, aged between 12 and 18, were involved in the UK workshop. One young person decided not to participate because of exam preparations, and another did not reply. The CYP who participated in the workshop attended the CYP's priority‐setting workshop for the Children's Cancer PSP in the United Kingdom. It is important to mention that the young people in the Dutch workshop had been involved in a JLA PSP in which adults have been involved as well. The young people involved in the UK workshop had been involved in a JLA PSP workshop in which only CYP were involved, while adults were involved in a separate workshop. The CYP involved in the workshops were all familiar with at least one of the workshop leaders, because of their earlier collaboration during the JLA workshops.

**Table 1:** Characteristics of the young people involved in the workshops.

	Workshop 1: Dutch group	Workshop 2: UK group
Children and young people	Female (13), second year of secondary school. Inflammatory bowel disease Female (14), not going to school because of disease. Inflammatory bowel disease Male (19), University of Applied Sciences‐Nursing. Inflammatory bowel disease Female (21), University, Psychology. Glycogen‐storage disease (individual interview)	Female (12), secondary school. Cancer. Female (15), secondary school. Sibling had cancer. Male (16), secondary school. Cancer. Female (18), sixth form. Cancer.

### 4.2 Results of the Mentimeter

**Figure 1:**
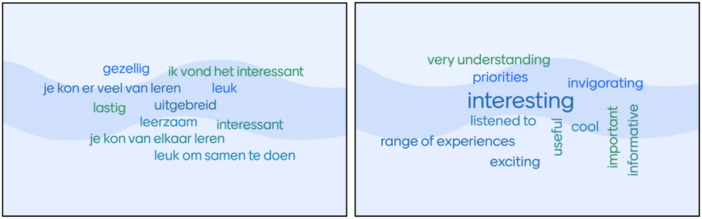
(a) Words that were associated with the James Lind Alliance according to the young people in the Dutch workshop (Dutch). (b) Words that were associated with the experiences of attending the Children's Cancer PSP priority setting workshop for CYP (UK workshop).

The following figures display the words that were entered in the Mentimeter during the Dutch and UK workshops (Figure 1a,b). The words are explained below the figures.

#### 4.2.1 Mentimeter Results From the Dutch Workshop

The young people entered 10 words: cosy, informative (3x), difficult, interesting (2x), pleasant, extensive and nice to collaborate. Six words were explored in more detail during the workshop, and a summary of the discussions for each word has been compiled.

The CYP evaluated the JLA process as:

- Informative—It was informative to gather tips and coping strategies from peers who share the same disease.
- Difficult—Agreeing the Top 10 research questions was challenging due to a lack of consensus within the group.
- Interesting—It was interesting to learn new things and to hear that other young people, physicians and researchers considered questions more important than expected.
- Pleasant—It was a pleasant experience to have the young people's voices heard instead of completing a survey where there is no evaluation of what happened with the results.
- Extensive—The approach was extensive and time‐consuming, requiring young people to concentrate for extended periods. However, the breaks in between made it manageable.
- Nice to collaborate—It was nice to collaborate with other young people and health professionals at the same time.

#### 4.2.2 Mentimeter Results From the UK Workshop

The young people entered 12 words to describe their experience of attending the priority setting workshop: very understanding, priorities, interesting (2x), listened to, range of experiences, exciting, invigorating, useful, cool, important and informative. Seven words were explored in more detail during the workshop, and a summary of the discussions for each word has been compiled.

The CYP evaluated their experience of attending the priority‐setting workshop as:

- Invigorating—it was ‘stimulating’ to hear about other people's experiences, this young person felt they had learned something from coming to the workshop, it was interesting to hear from other people.
- Exciting—it was exciting to be there, to be part of the workshop, to have a chance to get involved and have a say. It was also exciting to hear different people's experiences and thoughts.
- Listened to—everyone had a chance to say what they wanted to say, which was good, and now charities (funders) will know what children want research to focus on.
- Very understanding—this was linked to being ‘listened to’, everyone was accepting of what you wanted to say, everyone listened to each other and did not interrupt. There were not any right or wrong answers, you could say your opinions.
- Cool—it was cool to hear others’ experiences of cancer and to hear from people who had experienced cancer more recently as they remembered more about it and had some different experiences (such as being at school while having cancer).
- Useful—it was useful to find out what other people thought, everyone had different experiences and a wide range of opinions.
- Interesting—this word was mentioned many times when discussing the other words, it was interesting to hear different people's opinions and find out their experiences.

### 4.3 Tips From CYP

Tips from young people involved in the workshops are presented in Table 2. The tips are divided into three categories, to reflect the different elements of the JLA method: steering group, surveys and the final priority‐setting workshop including preparations and evaluations. Tips centred on three themes: making participants feel comfortable (14 tips); making the process accessible (9 tips); making the involvement meaningful (9 tips). The first theme focused on making participants feel comfortable by creating a welcoming and supportive environment where CYP feel at ease. This involves ensuring that CYP feel respected, valued and comfortable, which can enhance their willingness to participate and share their thoughts openly. The second theme considered making the process accessible, by removing barriers that might prevent people from participating fully. It includes providing clear instructions, using inclusive language, offering necessary accommodations and ensuring that the process is understandable and reachable for everyone involved. The third theme focused on making the involvement of young people meaningful, this involves actively listening to their perspectives, integrating their feedback into decision‐making processes, giving them opportunities to influence outcomes in a way that feels significant and rewarding to them and evaluating the involvement afterwards. Tips are presented as paraphrases from the CYP and some tips are accompanied with explanations given by the CYP. The numbering of the tips in the table has been chosen randomly, with no significance assigned to the importance of each tip. We opted to number the tips to make it easier to refer to different tips. Table 3 provides an overview of the tips, the themes they are categorized into, and the workshop in which the tips were mentioned.

**Table 2:** Tips from the young people involved in the workshops in the Netherlands and the United Kingdom.

Tips from the young people
Steering group
1. Have two groups, one for adults and professionals and one for children and young people but bring them together sometimes so that they can hear each other's views, interact and collaborate.
2. Allow time for young people and professionals to get to know each other and for professionals to explain their roles to the young people involved.
Survey
3. Make sure the survey looks interesting, use images and colour.
4. Online surveys are good for young people. Younger children (who are literate) may prefer to have the option to fill in a paper survey.
5. Sometimes it's more enjoyable to express your opinion through a workshop rather than a survey. However, a survey is quicker to fill out, so offer both options if possible.
6. Generating questions is hard but writing what is important to you is easier.
Ask participants to write what is important to them, do not ask them to send in their responses as questions.
7. Approach young people through social media, email, and their own health and care professionals.
For example, questionnaires could be distributed in the waiting room since the children and young people are already waiting there.
8. Try linking a reward to completing the survey.
9. Let the participants know what will happen to what they say in the survey, what the impact will be.
Final priority setting workshop—Preparing the workshop
10. Discuss with young people what time would be suitable for them to schedule the workshop.
11. The workshop should be planned in one day instead of multiple sessions.
This is easier for the children and young people as they have to travel only once.
12. Have an activity or questions for participants to answer before coming to the session, and then at the start everyone can share something they have already prepared.
This might help everyone to feel more confident as they have something ready to share, rather than having to think about what to say when they might be feeling nervous.
13. Ensure more than one child or young person is involved.
No one needs to feel alone. For example, being able to attend with an older sibling may be especially helpful for younger participants.
14. Involve siblings in the workshop.
The experiences of family members of someone with a health condition are also important.
15. Provide information about what will happen at the workshop, step by step, so participants know what to expect and can feel more prepared in advance for what they might like to say.
Final priority setting workshop—During the workshop
16. Play music as people arrive to help everyone feel a bit livelier and more open to sharing.
17. Allow time for everyone to get to know each other before moving on to discussing the questions as this will make it easier for people to share what they think.
18. Have a fun activity which involves moving around to start off.
19. Have colouring or a craft activity to do to occupy your hands during the discussion.
Sometimes people feel more confident to open up when they have something to do.
20. Ensure a person is involved that specifically monitors and advocates for the involvement of children and young people ensuring that they are able to express their opinions and listened to.
Children do not necessarily need to speak a lot, if their opinions are listened to that is what matters.
21. Reassure participants that everyone's opinions matter, there are no right or wrong answers, and no‐one will be judged.
Young people may worry more about being truthful and feel under pressure to ‘get it right’ and not give the ‘wrong’ responses.
22. Have all young people agree on a signal or word to use if they want to leave the room. Have some play stuff in the room, that they can then go to if feeling nervous.
23. Begin by having everyone explain why certain questions are important to them.
This way, everyone's motivation is clear, allowing you to empathize with them and making it easier to vote.
24. Make sure the questions being discussed are easy to understand and not too long.
It is easier to contribute if the questions are clear, and you do not have to think too much about what they mean.
25. Make sure the questions being discussed do not contain scary terms.
Sometimes professionals share a lot of information that young people might find daunting. Try to prevent this.
26. Ensure there is time planned for breaks.
During the breaks people might get to know each other better and this might increase the collaboration.
27. Offering a reward (or lunch) as a thank you for participation makes children and young people more likely to join in.
Final priority setting workshop—After the workshop
28. Follow up with everyone after the workshop and ask if there was anything young people thought of afterwards that they would like to add to the discussions.
Sometimes participants might think of something afterwards that they wished they had been able to add.
29. Evaluating the workshop is important. When asking for feedback after the workshop, make sure this is done soon afterwards, do not wait too long.

**Table 3:** Tips from the young people involved in the workshops in the Netherlands and the United Kingdom, categorized by the workshop in which the tips were mentioned and the themes into which the tips are categorized.

Tips from the young people		NL	UK		Theme
Steering group					
1. Have two groups, one for adults and professionals and one for children and young people but bring them together sometimes so that they can hear each other's views, interact and collaborate.					Comfort
2. Allow time for young people and professionals to get to know each other and for professionals to explain their roles to the young people involved.					Comfort
Survey					
3. Make sure the survey looks interesting, use images and colour.					Access
4. Online surveys are good for young people. Younger children (who are literate) may prefer to have the option to fill in a paper survey.					Access
5. Sometimes it's more enjoyable to express your opinion through a workshop rather than a survey. However, a survey is quicker to fill out, so offer both options if possible.					Access
6. Generating questions is hard but writing what is important to you is easier.					Access
Ask participants to write what is important to them, do not ask them to send in their responses as questions.					
7. Approach young people through social media, email, and their own health and care professionals.					Access
For example, questionnaires could be distributed in the waiting room since the children and young people are already waiting there.					
8. Try linking a reward to completing the survey.					Access
9. Let the participants know what will happen to what they say in the survey, what the impact will be.					Meaning
Final priority setting workshop—Preparing the workshop					
10. Discuss with young people what time would be suitable for them to schedule the workshop.					Access
11. The workshop should be planned in one day instead of multiple sessions.					Access
This is easier for the children and young people as they have to travel only once.					
12. Have an activity or questions for participants to answer before coming to the session, and then at the start everyone can share something they have already prepared.					Comfort
This might help everyone to feel more confident as they have something ready to share, rather than having to think about what to say when they might be feeling nervous.					
13. Ensure more than one child or young person is involved.					Comfort
No one needs to feel alone. For example, being able to attend with an older sibling may be especially helpful for younger participants.					
14. Involve siblings in the workshop.				Comfort
The experiences of family members of someone with a health condition are also important.					
15. Provide information about what will happen at the workshop, step by step, so participants know what to expect and can feel more prepared in advance for what they might like to say.				Comfort
Final priority setting workshop—During the workshop					
16. Play music as people arrive to help everyone feel a bit livelier and more open to sharing.				Comfort
17. Allow time for everyone to get to know each other before moving on to discussing the questions as this will make it easier for people to share what they think.					Comfort
18. Have a fun activity which involves moving around to start off.					Comfort
19. Have colouring or a craft activity to do to occupy your hands during the discussion.				Comfort
Sometimes people feel more confident to open when they have something to do.					
20. Ensure a person is involved that specifically monitors and advocates for the involvement of children and young people ensuring that they can express their opinions and are listened to.					Meaning
Children do not necessarily need to speak a lot, if their opinions are listened to that is what matters.					
21. Reassure participants that everyone's opinions matter, there are no right or wrong answers, and no‐one will be judged.				Comfort
Young people may worry more about being truthful and feel under pressure to ‘get it right’ and not give the ‘wrong’ responses.					
22. Have all young people agree on a signal or word to use if they want to leave the room. Have some play stuff in the room, that they can then go to if feeling nervous.					Comfort
23. Begin by having everyone explain why certain questions are important to them.				Meaning
This way, everyone's motivation is clear, allowing you to empathize with them and making it easier to vote.				
24. Make sure the questions being discussed are easy to understand and not too long.				Meaning
It is easier to contribute if the questions are clear, and you do not have to think too much about what they mean.					
25. Make sure the questions being discussed do not contain scary terms.					Comfort
Sometimes professionals share a lot of information that young people might find daunting. Try to prevent this.					
26. Ensure there is time planned for breaks.					Comfort
During the breaks people might get to know each other better and this might increase the collaboration.					
27. Offering a reward (or lunch) as a thank you for participation makes children and young people more likely to join in.					Access
Final priority setting workshop—After the workshop					
28. Follow up with everyone after the workshop and ask if there was anything young people thought of afterwards that they would like to add to the discussions.				Meaning
Sometimes participants might think of something afterwards that they wished they had been able to add.					
29. Evaluating the workshop is important. When asking for feedback after the workshop, make sure this is done soon afterwards, do not wait too long.				Meaning

## 5 Discussion

This project aimed to collect experiences and tips from CYP taking part in JLA PSPs and to develop guidance for the JLA on involving CYP in PSPs. Results provide a list of 29 tips for involving CYP in JLA PSPs. Tips were grouped into five categories: steering group, survey, final priority setting workshop—preparing the workshop, final priority setting workshop—during the workshop and final priority setting workshop—after the workshop. Tips centred on making participants feel comfortable, making the process accessible and making the involvement of CYP meaningful. This list of 29 tips for involving CYP in JLA PSPs guides future partnerships seeking to involve CYP. It guides teams during the planning, execution and completion of the project. The following sections provide illustrative examples of the JLA PSPs conducted by the authors of this project, demonstrating how the recommendations provided by the CYP can be integrated into the process.

### 5.1 Making Participants Feel Comfortable

The young people emphasized the importance of allocating sufficient time and attention to the formation of a supportive and comfortable group dynamic. This was paramount at every stage of the JLA PSP, from the initial meetings of the steering group to the final priority‐setting workshop. The young people gave examples of what to do to make them feel comfortable, for example, preparing a question at home, so they can share something rather than having to think about what to say when they might be feeling nervous, reassuring the CYP that everyone's opinions matter, there are no right or wrong answers and no‐one will be judged. Another tip that CYP gave in both the Dutch and UK workshop was to have two steering groups, one for adults and professionals and one for CYP but bring them together so they can hear each other's views, interact and collaborate. The Children's Cancer PSP [26] and the Juvenile Idiopathic Arthritis PSP [28] are examples of PSPs in which the CYP and adults participated in separate workshops. They chose not to combine the different groups but presented the priorities listed as important by CYP during the workshop in which the adults participated. Future PSPs could adopt an approach, wherein the CYP workshop and the adult workshop occur on the same day, with the groups being integrated at the end of the day.

### 5.2 Making the Process Accessible

Children and young people highlighted that expressing opinions through workshops can be more enjoyable than completing surveys. They also stated that filling out a survey is quicker. Therefore, it is beneficial to offer both options if feasible. To gain insight from a child's perspective, Aussems and colleagues conducted focus groups with children living with juvenile arthritis to jointly develop and prioritize research questions, rather than relying on the traditional online survey methodology. This approach was adopted to address the under‐representation of children in surveys [28].

CYP suggested planning the workshop in a single day rather than multiple sessions, as this is more convenient for them, reducing the need for multiple journeys. Time constraints for maximising the involvement of CYP are essential, and adjustments to do so might need to be made. For example, Aussems and colleagues developed a Top 5 research uncertainties instead of the JLA's usual Top 10 [28, 31]. This turned out to be successful as a Top 10 would have required more discussion time, which would have resulted in an additional session due to the limited concentration levels of the children. Aldiss and colleagues also adopted this approach [25].

CYP mentioned that ensuring the research material (surveys, invitations, etc.) looks interesting is important. The Children's Cancer PSP used age‐appropriate animations to reach children and help them understand the project and what they were asked to do. Two animations were produced, one for younger (https://www.youtube.com/watch?v=O492QZ1myko&t=72s), and one for older children (https://www.youtube.com/watch?v=pRaRuMr7ol0), children and families could self‐select the animation that looked most applicable to them. Another adjustment could be made regarding the survey, the Children's Cancer and the Paediatric IBD PSP made multiple versions of the surveys, aimed at children of different ages (4–7 years, 8–12 years, 13–15 years and 16 years or older). The surveys varied in the complexity of language used in the introduction section and questions [25]. In addition, CYP participating in the steering group of the Paediatric IBD PSP designed flyers themselves for the different age groups, containing information and animations to encourage CYP to fill out the surveys [27].

### 5.3 Making the Involvement of CYP Meaningful

The JLA PSP about juvenile idiopathic arthritis did not include children in the final priority workshop, as the project team felt that younger children would not have an equal say. The young adult patients assumed a role of advocacy for the children during the final workshop. This serves as an illustrative example of the fact that there are alternatives for children to express themselves for their opinions to have influence during this phase. It is sufficient that their views are listened to and taken seriously. In this instance, the young adult patients ensured that the younger patients’ voices were heard [28, 31]. Similarly, children did not attend the final priority setting workshop for the Children's Cancer PSP, a separate workshop had been held with children to determine their top priorities. When the adult participants at the final workshop were told which of the questions featured in the children's Top 5 priorities, often they moved up in the ranking and were reflected in the final Top 10 priorities for Children's Cancer [26]. The paediatric IBD PSP, however, decided to bring CYP, caregivers and healthcare professionals together in the final workshop. The workshop leaders ensured that all participants had equal voices and specifically paid attention to the perspectives and needs of the children [27].

In addition to guiding the JLA PSP, insights were also shared regarding post‐event activities, with a particular focus on ensuring that the involvement of CYP has had an impact. Peeks and colleagues, who were responsible for the JLA PSPs about Glycogen Storage Diseases (GSD), discussed the strategy for dissemination to reach the stakeholders after the final workshop so they know what impact they made. The dissemination strategy included the following elements: a detailed scientific manuscript, a lay report, a social media and website campaign, press releases via patient organisations and channels from the JLA and planned activities at the International GSD Conference [32].

### 5.4 Reflection and Comparison to Existing Literature

The young people were generally positive about the JLA method for developing a research agenda together. They felt listened to during the JLA workshops they had attended. During the workshops, it was noticeable that the young people often agreed with each other and tended to complement each other's points rather than extensively debate the different topics. At times, specially for some younger participants, it was quite challenging to reflect on the process and precisely articulate what they found effective or what could be improved. In addition, we thought it was easier for the young people to discuss elements of the JLA method they had experience with, but they did still have some tips for other elements, they just were not as detailed. The authors of this article encourage future teams, that involve CYP and use the tips, to reflect on their collaboration to ensure the tips are evaluated and updated.

More and more studies have been conducted on how to involve children in research, updated by several Young People's Advisory Groups (YPAG) and the members of the INVOLVE CYP's working group, which includes young people [33]. They state that involvement should start as early as possible in the research process and do not make assumptions about children's capabilities. Furthermore, Alderson and colleagues co‐developed tips for involving vulnerable young people, such as looked after children [34]. They highlighted that looked after children can be involved in research if researchers have enough time, resources and willingness to work at the pace of the children involved. The recurring message that these studies consistently endeavour to communicate is that the involvement of CYP matters, they have unique insights into their lives, and we need to tailor the way we work with them, whether for consultation or for conducting research with them [33, 34]. Involving CYP in the development of our tips has once again confirmed that they can provide valuable input. Their unique perspectives and insights have significantly enriched the process, demonstrating their capability and the importance of their contributions. Additionally, this involvement has fostered a sense of respect and appreciation, as almost every young person we approached was willing to participate and offer their input, despite their often already busy schedules. This dedication highlights their commitment and we believe it is essential to handle their time with care. Therefore, the project group is committed to implementing these tips to ensure that the involvement of young people has a real impact on future projects.

### 5.5 Implementation

The tips are published on the JLA website and linked to the JLA guidebook making them openly available to everyone (https://www.jla.nihr.ac.uk/documents/involving-children-and-young-people-james-lind-alliance-priority-setting-partnerships). Involving CYP remains tailor‐made therefore involving them in deciding which tips to use and which not is still important. The list of tips can be presented to the CYP, and they can agree or disagree together on which of the tips they want to implement. In this case, CYP are truly involved in decision‐making from the beginning of the JLA PSP. While the tips originate from CYP and are specifically designed for PSPs involving CYP, some of them may also apply to PSPs involving only adults. For example, ‘Let the participants know what will happen to what they say in the survey, what the impact will be’, ‘Play music as people arrive to help everyone feel a bit livelier and more open to sharing.’, or ‘Do not wait too long when asking for feedback after the workshop’. By listening to and acting upon the tips of CYP, researchers and organisations can create more relevant, inclusive and impactful research agendas. We specifically invited CYP who had already taken part in a JLA PSP, and by now they were 12 years old or older. When it comes to inviting children under 12 to work together on research agenda setting, we think this is certainly important as well, as they also have the right to be listened to and taken seriously. They can provide insight to what their most important questions are, as they also have daily issues regarding their medical condition that they can share insights about. We understand that a younger age group would also require an approach that enables their meaningful participation, based on their capabilities, as the way they express their questions may be different. We believe the developed list contains advice that is applicable for all CYP including the tip to ‘ensure that more than one child or young person is involved. No one needs to feel alone. For example, being able to attend with an older sibling may be especially helpful for younger participants’. Furthermore, in the juvenile idiopathic arthritis PSP children aged between 8 and 12 were involved. The authors published guidance on how to involve the younger children in JLA PSPs on the JLA website (https://www.jla.nihr.ac.uk/news/involving-children-setting-priorities-research-pediatric-diseases).

### 5.6 Strengths and Weaknesses

The involvement of young people is a strength of this project. The project group recognised early in the project that developing guidance based only on their opinions, without the direct input of young people who had experienced being involved in a PSP, might result in guidance that was less useful and did not truly reflect how to best involve CYP. Indeed, the tips from young people contain insightful suggestions that the group had not considered, such as having items available to occupy your hands during discussions and bringing something preprepared to share at the start of the workshop. As we involved young people with experience in a JLA PSP workshop exclusively for young people, as well as those with experience in a JLA PSP where both young people and adults were involved, the tips provided can apply to both scenarios: JLA PSP workshops that separate young participants from adults and those that involve both groups together.

It is important to mention as a weakness that the young people were involved in specific elements of the PSP process. Most of them did not participate in the whole PSP process. Furthermore, even though we strived to involve CYP in research agendas developed with different methods, we only managed to include CYP involved in JLA PSPs. Young people who have been involved in shaping research agendas using different methods might have offered additional tips or insights. This limitation was due to the higher availability and accessibility of participants with JLA experience. A previous study showed that even though there are more approaches to developing a research agenda together with CYP, the JLA approach is the most frequently used method [22]. No CYPs involved in the juvenile idiopathic arthritis PSP were at the Dutch workshop. Although there is significant interest in how researchers can approach children to involve them in research, this was not within the scope of the present study. Our focus was on enhancing their involvement in the research process itself.

### 5.7 Conclusion

This list of 29 tips for involving CYP in JLA PSPs guides future partnerships seeking to involve CYP. Involving CYP does take additional time and consideration, to ensure that they are involved in a way that best suits them. These tips which have come directly from young people indicate their suggestions on how to involve CYP in a way that is comfortable, accessible and meaningful. Children and young people have a right to be consulted on matters that affect them. We therefore encourage anyone undertaking a research priority‐setting exercise which is on a topic that impacts CYP to involve CYP at all stages, including suggesting and prioritising research questions. This will ensure that the resulting priorities reflect more widely the views of those with lived experience, rather than only the views of adults.

## Author Contributions

**Laura Postma:** formal analysis, investigation, resources, visualization, writing ‐ original draft preparation. **Faith Gibson:** investigation, project administration, resources, writing ‐ review and editing. **Jasmijn Z. Jagt:** investigation, resources, writing ‐ review and editing. **Karijn Aussems:** resources, writing ‐ review and editing. **Helen Barrett:** funding acquisition, writing ‐ review and editing. **Malou Luchtenberg:** supervision, writing ‐ review and editing. **Casper G. Schoemaker:** resources, writing ‐ review and editing. **Rachel K. Temple:** writing ‐ review and editing. **Susie Aldiss:** investigation, project administration, resources, writing ‐ review and editing.

## Ethics Statement

The authors have nothing to report.

## Conflicts of Interest

The authors declare no conflicts of interest.

## Data Availability

The data that support the findings of this project are available on request from the corresponding author. The data are not publicly available due to privacy or ethical restrictions.
